# Supplementary material for: Crawling and Gliding: A Computational Model for Shape-Driven Cell Migration
Source: PLoS Comput Biol. 2015 Oct 21;11(10):e1004280. doi: 10.1371/journal.pcbi.1004280 (PMC4619082; doi:10.1371/journal.pcbi.1004280)
Supplement: S1 Code — (ZIP) [file pcbi.1004280.s012.zip › release/tst/doc/html/graph_legend.html]

Tissue Simulation Toolkit: Graph Legend


Tissue Simulation Toolkit
 0.1.4.1


- Main Page
- Namespaces
- Classes
- Files

Graph Legend

This page explains how to interpret the graphs that are generated by doxygen.

Consider the following example:

/\*! Invisible class because of truncation \*/

class Invisible { };

/\*! Truncated class, inheritance relation is hidden \*/

class Truncated : public Invisible { };

/\* Class not documented with doxygen comments \*/

class Undocumented { };

/\*! Class that is inherited using public inheritance \*/

class PublicBase : public Truncated { };

/\*! A template class \*/

template<class T> class Templ { };

/\*! Class that is inherited using protected inheritance \*/

class ProtectedBase { };

/\*! Class that is inherited using private inheritance \*/

class PrivateBase { };

/\*! Class that is used by the Inherited class \*/

class Used { };

/\*! Super class that inherits a number of other classes \*/

class Inherited : public PublicBase,

protected ProtectedBase,

private PrivateBase,

public Undocumented,

public Templ<int>

{

private:

Used \*m\_usedClass;

};

This will result in the following graph:


The boxes in the above graph have the following meaning:

- A filled gray box represents the struct or class for which the graph is generated.
- A box with a black border denotes a documented struct or class.
- A box with a grey border denotes an undocumented struct or class.
- A box with a red border denotes a documented struct or class forwhich not all inheritance/containment relations are shown. A graph is truncated if it does not fit within the specified boundaries.

The arrows have the following meaning:

- A dark blue arrow is used to visualize a public inheritance relation between two classes.
- A dark green arrow is used for protected inheritance.
- A dark red arrow is used for private inheritance.
- A purple dashed arrow is used if a class is contained or used by another class. The arrow is labeled with the variable(s) through which the pointed class or struct is accessible.
- A yellow dashed arrow denotes a relation between a template instance and the template class it was instantiated from. The arrow is labeled with the template parameters of the instance.


---

Generated on Thu Aug 14 2014 22:04:01 for Tissue Simulation Toolkit by  

 1.8.6
